# Four novel *Cit7GlcTs* functional in flavonoid 7-*O*-glucoside biosynthesis are vital to flavonoid biosynthesis shunting in citrus

**DOI:** 10.1093/hr/uhae098

**Published:** 2024-04-25

**Authors:** Ziyu Yuan, Gu Li, Huixian Zhang, Zhaoxin Peng, Wenyu Ding, Huan Wen, Hanxin Zhou, Jiwu Zeng, Jiajing Chen, Juan Xu

**Affiliations:** National Key Laboratory for Germplasm Innovation and Utilization of Horticultural Crops, College of Horticulture and Forestry, Huazhong Agricultural University, Wuhan 430070, China; Hubei Hongshan Laboratory, Wuhan 430070, China; National Key Laboratory for Germplasm Innovation and Utilization of Horticultural Crops, College of Horticulture and Forestry, Huazhong Agricultural University, Wuhan 430070, China; Hubei Hongshan Laboratory, Wuhan 430070, China; National Key Laboratory for Germplasm Innovation and Utilization of Horticultural Crops, College of Horticulture and Forestry, Huazhong Agricultural University, Wuhan 430070, China; Hubei Hongshan Laboratory, Wuhan 430070, China; National Key Laboratory for Germplasm Innovation and Utilization of Horticultural Crops, College of Horticulture and Forestry, Huazhong Agricultural University, Wuhan 430070, China; National Key Laboratory for Germplasm Innovation and Utilization of Horticultural Crops, College of Horticulture and Forestry, Huazhong Agricultural University, Wuhan 430070, China; National Key Laboratory for Germplasm Innovation and Utilization of Horticultural Crops, College of Horticulture and Forestry, Huazhong Agricultural University, Wuhan 430070, China; National Key Laboratory for Germplasm Innovation and Utilization of Horticultural Crops, College of Horticulture and Forestry, Huazhong Agricultural University, Wuhan 430070, China; Key Laboratory of South Subtropical Fruit Biology and Genetic Resource, Institute of Fruit Tree Research, Guangdong Academy of Agricultural Sciences, Guangzhou 510640, China; National Key Laboratory for Germplasm Innovation and Utilization of Horticultural Crops, College of Horticulture and Forestry, Huazhong Agricultural University, Wuhan 430070, China; Hubei Hongshan Laboratory, Wuhan 430070, China; National Key Laboratory for Germplasm Innovation and Utilization of Horticultural Crops, College of Horticulture and Forestry, Huazhong Agricultural University, Wuhan 430070, China; Hubei Hongshan Laboratory, Wuhan 430070, China

## Abstract

Citrus fruits have abundant flavonoid glycosides (FGs), an important class of natural functional and flavor components. However, there have been few reports about the modification of UDP-glycosyltransferases (UGTs) on flavonoids in citrus. Notably, in flavonoid biosynthesis, 7-*O*-glucosylation is the initial and essential step of glycosylation prior to the synthesis of flavanone disaccharides, the most abundant and iconic FGs in citrus fruits. Here, based on the accumulation of FGs observed at the very early fruit development stage of two pummelo varieties, we screened six novel flavonoid 7-*O*-glucosyltransferase genes (*7GlcTs*) *via* transcriptomic analysis and then characterized them *in vitro*. The results revealed that four Cg7GlcTs possess wide catalytic activities towards various flavonoid substrates, with CgUGT89AK1 exhibiting the highest catalytic efficiency. Transient overexpression of *CgUGT90A31* and *CgUGT89AK1* led to increases in FG synthesis in pummelo leaves. Interestingly, these two genes had conserved sequences and consistent functions across different germplasms. Moreover, *CitUGT89AK1* was found to play a role in the response of citrus to Huanglongbing infection by promoting FG production. The findings improve our understanding of flavonoid 7-*O*-glucosylation by identifying the key genes, and may help improve the benefits of flavonoid biosynthesis for plants and humans in the future.

## Introduction

Citrus fruits are extensively consumed and cultivated on a global scale [1]. In 2021, the global citrus fruit production reached about 13.9 million tons, with China accounting for 42.88% of the total production (FAO, https://www.fao.org). Besides fresh consumption, citrus fruits are also processed into juice, cans, and some pharmaceutical materials [2]. For example, ‘Huazhou’ pummelo (*C. grandis* var. ‘Tomentosa’) is the source material of traditional Chinese medicine ‘Huajuhong’, which has been used as an antitussive and expectorant in clinic for thousands of years due to its particularly high content of flavonoids [3]. In recent years, there has been continuously increasing demand of the consumers for food products with medical and health-promoting properties, which has greatly stimulated research initiatives to explore the functional components in different foods, such as flavonoids, carotenoids, vitamin C, minerals, and dietary fiber. Flavonoids are dominant secondary metabolites in citrus fruits [4]. According to their structural characteristics, flavonoids in plants can be generally categorized into flavanones, flavones, isoflavones, flavonols, flavanols, and anthocyanidins [5], which contribute to the formation of pigments [6] and flavors [7], and play crucial roles in defense against various biotic [8, 9] and abiotic [10–12] stresses.

Citrus flavonoids are mostly glycosyl derivatives, which confer them with high stability, solubility, and bioactivity [13]. Citrus fruits are known to contain high content of flavonoid glycosides (FGs), particularly ‘Huazhou’ pummelo, whose FGs level can reach 256.48 mg/g and account for over 25% of the fruit dry weight [14]. Recently, FGs have attracted increasing attention due to their diverse beneficial effects to human health. For example, naringin is the main pharmacological component of ‘Huajuhong’, which has various positive effect on bone regeneration, metabolic syndrome, oxidative stress response, and central nervous system diseases [15]. Furthermore, FGs also play certain roles in plant defense against various diseases, such as citrus canker [16] and Huanglongbing (HLB) [17, 18]. ‘Valencia’ oranges with HLB infection exhibit elevated concentrations of flavonoids in the peel, pulp, and juice, including the FGs hesperidin and narirutin [19]. Grapefruits with HLB infection significantly produce naringin [20]. Therefore, it is particularly important to investigate the biosynthesis of FGs so as to better utilize citrus flavonoids for human welfare and plant health.

Production of plant flavonoids is an important branch of the phenylpropanoid metabolic pathway. The process is initiated with the enzymatic catalysis of the precursor phenylalanine by various enzymes, including phenylalanine ammonia-lyase (PAL), cinnamate 4-hydroxylase (C4H), 4-coumarate: CoA ligase (4CL), chalcone synthase (CHS), and chalcone isomerase (CHI), resulting in the formation of naringenin, which serves as the intermediate precursor for flavonoid synthesis [4]. In citrus, naringenin is mostly modified by oxygenmethylase or hydroxylase to form other flavanone aglycones (hesperetin, eriodictyol or isosakuranetin) [21]. Additionally, naringenin can also be catalyzed by flavone synthase (FNS) and flavonol synthase (FLS) to form flavone and flavanol aglycones, respectively. Then, the above flavonoid aglycones are mostly glucosylated at the 7-position by flavonoid 7-*O*-glucosyltransferase (7GlcT) to form corresponding flavonoid 7-*O*-glucosides, which are further glycosylated by either 1,2-rhamnosyltransferase (1,2RhaT) to yield bitter flavonoid neohesperidosides or 1,6-rhamnosyltransferase (1,6RhaT) to generate non-bitter flavonoid rutinosides [22] (Fig. S1). Both flavonoid neohesperidosides and rutinosides are flavonoid disaccharides, with flavanone disaccharides as the most abundant and iconic FGs as the end products in citrus fruits. Previous research has documented the existence of two functional UDP-glycosyltransferases, namely Cm1,2RhaT [23] and Cs1,6RhaT [7], from pummelo and sweet orange, respectively. These enzymes play vital roles in the formation of flavonoid disaccharides, the predominant and characteristic metabolites in the flavonoid profile of pummelo or sweet orange. Although it is clear that production of flavonoid 7-*O*-glucosides is the initial step in the biosynthesis of flavonoid disaccharides in citrus, there have been few reports on the genes governing this crucial process.

Flavonoid 7GlcT belong to the UGT family and have catalytic activity on the 7-hydroxy position of flavonoid substrates. To date, there have been many reports on flavonoid 7GlcT in different plants. For example, At7GlcT can take flavonols as the substrate to produce 7-*O*-glucosides in *Arabidopsis thaliana* [24]. In tea (*Camellia sinensis*), CsUGT75L12 specifically transports the glucose unit from UDP-glucose to the 7-hydroxy position of the flavonoid to produce 7-*O*-glucoside [25]. Sb7GlcT can catalyze baicalein to produce baicalein 7-*O*-glucoside in hairy roots in response to wounding or salicylic acid treatments in the traditional Chinese medicine *Scutellaria baicalensis* [26]. CsUGT76F1 has been reported to catalyze flavonoids at the 7-hydroxy position in sweet orange, with special preference to catalyze flavonols [27]. In general, most 7GlcTs currently characterized are for flavonol or flavone substrates, and there have been few reports on 7GlcTs involved in the flavanone biosynthetic pathway. Recently, two CgUGTs with the functions of flavonoid 7-*O*-glucosyltransferase were identified in mature pummelo fruits *via* mGWAS analysis [28]. However, as flavonoid accumulation usually occurs at the early stage of citrus fruit development, but tends to decline after 90 days after flowering (DAF) [29], there may be some other 7GlcT members involved in the synthesis of citrus FGs, which may have greater contributions to the accumulation of flavonoids in citrus fruit before 90 DAF. Thus, identification of key citrus 7GlcTs is crucial for determining the shunting of downstream FGs so as to better understand the biosynthetic patterns of FGs in citrus.

In this study, an integrated analysis of transcriptomics and metabolomics was applied to mine candidate *7GlcTs* from pummelo (*C. grandis*). Functional analysis revealed that four out of the six Cg7GlcTs candidates were functional in glucosylation of the 7-hydroxy position to various flavonoid substrates. Transient overexpression in pummelo leaves demonstrated the effect of *CgUGT90A31* and *CgUGT89AK1* to promote the accumulation of FGs. We also found that the homologous genes of *CgUGT90A31* and *CgUGT89AK1* in *Poncirus trifoliata* had a 7-*O*-glucosylation function as well. In addition, HLB infection upregulated the expression of *CitUGT89AK1* and the accumulation of FGs in pummelo and loose skin mandarin. Overall, this study screened and characterized the key genes governing the initial step of glucosylation, which may improve our understanding of the flavonoid biosynthetic pathway in citrus and facilitate the tuning of flavonoid biosynthesis for plant defense and human health benefits.

## Results

### FGs constitute the primary metabolites of flavonoids in citrus fruits

**Figure 1:**
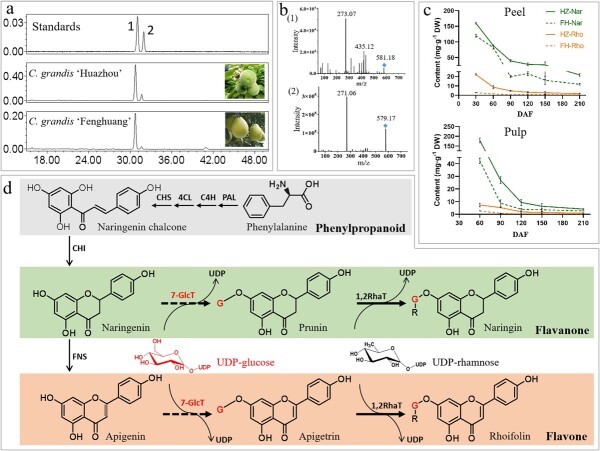
Identification, accumulation pattern, and speculation of biosynthetic pathways of FGs in pummelo fruits. (A) and (B) FGs in the pulp of ‘Huazhou’ pummelo and ‘Fenghuang’ pummelo fruit at 90 DAF. Peak 1 is naringin, and peak 2 is rhoifolin. (C) Dynamic changes in naringin and rhoifolin content throughout fruit development. Since fruit at 30 DAF had no pulp, the whole fruit was investigated as the peel; HZ, ‘Huazhou’ pummelo; FH, ‘Fenghuang’ pummelo; Nar, naringin; Rho, rhoifolin. (D) Biosynthetic pathway of FG compounds in pummelo. G, glucose; R, rhamnose.

To investigate the accumulation pattern of FGs in citrus fruits, we analyzed the FG profiles in mature fruit pulp of five representative citrus species. The HPLC results showed that there were eight kinds of dominant flavanone disaccharides in citrus fruits (Fig S2), and the accumulation of FGs in the pulp had certain species specificity. In addition, some FGs such as rhoifolin were also present in small amounts in citrus fruit pulp, which constitute stable FG profiles together with the dominant FGs in citrus. To further reveal the accumulation pattern of FGs during citrus fruit development, we selected ‘Huazhou’ pummelo (*C. grandis*) and ‘Fenghuang’ pummelo (*C. grandis*) fruits at distinct developmental stages as the experimental materials. HPLC elution profiles indicated that there were two FGs in the fruits (Fig. 1A), namely naringin (compound 1) and rhoifolin (compound 2) respectively belonging to flavanones and flavones, based on comparison of their retention time and ion fragmentation patterns with those of the standards (Fig. 1B). The levels of naringin and rhoifolin were the highest at the initial phase of the peel at 30 DAF or the pulp at 60 DAF, followed gradual decreases (Fig. 1C). Significantly, 7-*O*-glucosylation was the first step in transforming naringenin and apigenin into the final disaccharide derivatives of naringin and rhoifolin in pummelo, respectively (Fig. 1D). And Cit7GlcTs might play a key role in this process.

### Identification of putative *Cit7GlcTs* based on the pummelo genome

**Figure 2:**
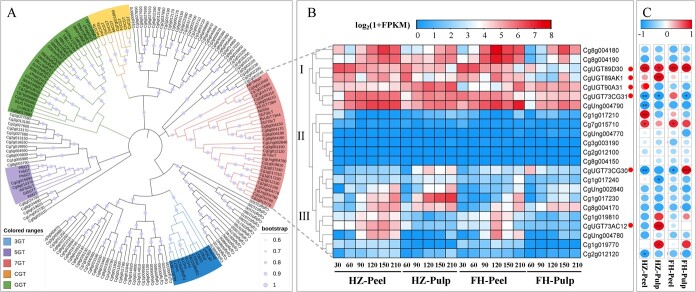
Identification of putative *7GlcTs* based on the pummelo genome. (A) Phylogenetic analysis of pummelo UGTs with other plant UGTs. Only bootstrap values > 60% are displayed, and each value is visualized using a light blue circle, whose size represents the corresponding metadata value in the middle of each branch. (B) Transcript level of the *7GlcTs* cluster in HZ and FH pummelo fruits at different developmental stages, red circles mark the genes screened for this article. (C) Correlation between the expression of *7GlcTs* and FG contents throughout fruit development. ^*^P < 0.05, ^**^P < 0.01, ^***^P < 0.001.

To mine possible *Cit7GlcTs* involved in the synthesis of FGs in citrus, we firstly annotated the *UGT* genes from the genome of *C. grandis*. Subsequently, a search was conducted for conserved domains of plant secondary product glycosyltransferase (PSPG) box, yielding 144 potential UGTs and 149 transcript sequences. The predicted UGTs had lengths ranging from 214 to 679 amino acids (aa), molecular weights ranging from 22.9 to 76.9 kDa, and pI values ranging from 5.11 to 9.18 (Table S1). Then, a phylogenetic tree was constructed by alignment of the full-length amino acid sequences with those of functionally characterized UGTs from citrus and other plants (Fig. 2A). The phylogenetic tree comprised five distinct groups, including 3-*O*-glycosyltransferases (3GTs), 5-*O*-glycosyltransferases (5GTs), 7-*O*-glycosyltransferases (7GTs), *C*-glycosyltransferases (CGTs), and glycoside glycosyltransferase (GGTs). As a result, 23 possible UGT sequences were clustered in the 7GTs group, which could be preliminarily identified as candidate genes encoding citrus flavonoid 7GlcTs.

The Cit7GlcT enzymes play a pivotal role in the initial step to catalyze the formation of FGs in citrus. We analyzed the transcription levels of 23 members from the 7GTs group in the ‘Huazhou’ pummelo and ‘Fenghuang’ pummelo fruit at different developmental stages. According to the transcription levels, candidate *7GlcTs* could be divided into three types: type I genes had high expression levels throughout the development; type II genes had low expression levels throughout the development; and type III genes only had high expression in some specific periods (Fig. 2B). Furthermore, we identified candidate *7GlcTs* involved in the accumulation of flavonoids through correlation coefficient (Pearson r) analysis between the transcription levels of *7GlcTs* and the contents of FGs (sum of naringin and rhoifolin). According to the screening criteria of correlation coefficient > 0.6 and the gene expression level of FPKM > 10 in at least one developmental stage, two (*Cg7g015690* and *Cg2g006310*) and three genes (*Cg7g015690, Cg5g012040,* and *Cg8g004210*) were found in the peel and pulp of ‘Huazhou’ pummelo, respectively; in addition, three genes (*Cg2g012130, Cg7g015690, Cg1g019780*) in peel and pulp of ‘Fenghuang’ pummelo were found as candidates (Fig. 2C). Therefore, a total of six *7GlcTs* were screened, including *Cg2g012130* (*CgUGT73CG30*), *Cg7g015690* (*CgUGT89D30*), *Cg2g006310* (*CgUGT90A31*), *Cg1g019780* (*CgUGT73CG31*), *Cg5g012040* (*CgUGT89AK1*), and *Cg8g004210* (*CgUGT73AC12*), named by the UGT Nomenclature Committee. The full-length sequences of six *Cg7GlcTs* were isolated from the cDNA library of ‘Huazhou’ pummelo pulp, and the sequencing analysis revealed that each Cg7GlcT contained a PSPG box that comprises 44 aa with glutamine (Q) as the last amino acid, which has been reported as the key residue that determines whether UGTs utilize UDP-glucose as a sugar donor [30, 31] (Fig. S3).

### Candidate Cg7GlcTs show broad-spectrum 7-hydroxy glucosylation properties *in vitro*

The protein encoded by the six Cg7GlcTs was expressed as a recombinant protein fused with maltose-binding protein tags, and the recombinant proteins were approximately 100 kDa as indicated by SDS–PAGE (Fig. S4). Hence, the enzymatic activity of Cg7GlcT recombinase on three distinct types of flavonoids (flavanones, flavones, and flavonols) was determined *in vitro*. Glucosylated products were identified by comparing the retention time and MS/MS data with those of the authentic compounds. When the commercial standards for predicted products were not available, their identities were inferred based on the results of analogous substrates and mass spectra (Fig. S5).

**Figure 3:**
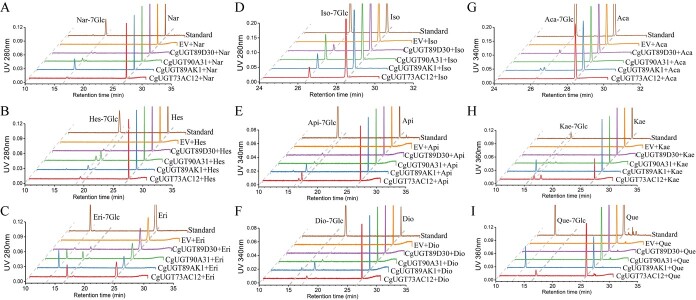
HPLC chromatograms of flavonones, flavones, and flavonols glucosylated by Cg7GlcTs *in vitro*. (A-D) Flavonone substrates: Nar, naringenin; Nar-7Glc, naringenin 7-*O*-glucoside; Hes, hesperetin; Hes-7Glc, hesperetin 7-*O*-glucoside; Eri, eriodictyol; Eri-7Glc, eriodictyol 7-*O*-glucoside; Iso, isosakuranetin; Iso-7Glc, isosakuranetin 7-*O*-glucoside. (E-G) Flavone substrates: Api, apigenin; Api-7Glc, apigenin 7-*O*-glucoside; Dio, diosmetin; Dio-7Glc, diosmetin 7-*O*-glucoside; Aca, acacetin; Aca-7Glc, acacetin 7-*O*-glucoside. (H-I) Flavonol substrates: Kae, kaempferol; Kae-7Glc, kaempferol 7-*O*-glucoside; Que, quercetin; Que-7Glc, quercetin 7-*O*-glucoside. MS/MS data of products and their authentic compounds are indicated in Fig. S5.

When using four flavanones (naringenin, hesperetin, eriodictyol and isosakuranetin) as substrates, CgUGT89D30, CgUGT90A31, CgUGT89AK1, and CgUGT73AC12 exhibited catalytic activities for 7-*O*-glucosylation on flavanones (Fig. 3A-D). Nevertheless, when hesperetin was used as the substrate, only CgUGT90A31 generated an additional peak resembling the mass spectra of hesperidin 7-*O*-glucoside, which might be one of its isomers. Similarly, when eriodictyol was used as the substrate, CgUGT89D30, CgUGT90A31, and CgUGT73AC12 yielded additional peaks that might be eriodictyol 7-*O*-glucoside isomers as indicated by the mass spectra data. Then, three flavones (apigenin, diosmetin, and acacetin) were fed in the reaction system, respectively (Fig. 3E-G). As a result, CgUGT89D30, CgUGT90A31, CgUGT89AK1, and CgUGT73AC12 could convert apigenin into apigenin 7-*O*-glucoside. However, CgUGT73AC12 predominantly produced an isomer of apigenin monoglucoside. In addition, CgUGT90A31, CgUGT89AK1, and CgUGT73AC12 demonstrated catalytic activity to produce diosmetin 7-*O*-glucoside. When using acacetin as the substrate, the above four Cg7GlcTs exhibited weak 7-*O*-glucosylation activities. When the substrates were kaempferol and quercetin, the most representative flavonol aglycones in citrus (Fig. 3H-I), CgUGT89AK1 exhibited no 7-*O*-glucosylation activity on kaempferol, whilst kaempferol monoglucoside other than the 7-position glucoside was formed. However, when the substrate was quercetin, CgUGT89AK1 showed a robust catalytic ability for 7-*O*-glucosylation, whereas CgUGT73AC12 exhibited glucosylation functions at other positions. As for CgUGT73CG30 and CgUGT73CG31, no flavonoid 7-*O*-glucosylation activity was detected (Fig. S6).

The product content comparison showed that when feeding with the above nine flavonoid substrates (Table 1), CgUGT90A31 had the most pronounced catalytic activity towards hesperetin and isosakuranetin; CgUGT73AC12 displayed the highest catalytic activity towards kaempherol; and CgUGT89AK1 exhibited the most pronounced enzymatic activity towards the remaining six substrates. These results indicated that the four Cg7GlcTs possess versatile catalytic capabilities towards various flavonoid substrates.

**Table 1:** Flavonoid 7-*O*-glucoside products from the catalysis of Cg7GlcTs on different types of flavonoid substrates

**Subclass**	**Substrate applied**	**Structure**		**Substituent group**	**Reaction product**	**[M+H]** ^**+**^	**CgUGT89D30**	**CgUGT90A31**	**CgUGT89AK1**	**CgUGT73AC12**	
							**product content (μM)** ^**a**^	**product content (μM)**	**product content (μM)**	**product content (μM)**	
**Flavanone**	Naringenin	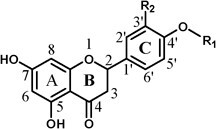		R1=H, R2=H	Nar 7-*O*-Glc	435.13	2.27 ± 0.53c	7.98 ± 1.35b	**17.06 ± 0.34a** ^**b**^	3.09 ± 0.48c	
	Hesperetin			R1=CH3, R2=OH	Hes 7-*O*-Glc	465.33	3.42 ± 0.11c	**11.36 ± 0.51a**	6.47 ± 0.68b	4.13 ± 2.05c	
	Eriodictyol			R1=H, R2=OH	Eri 7-*O*-Glc	451.12	2.07 ± 0.12b	14.09 ± 0.95a	**16.61 ± 2.71a**	2.45 ± 0.48b	
	Isosakuranetin				R1=CH3, R2=H	Iso 7-*O*-Glc	449.11	6.80 ± 1.77d	**18.84 ± 0.07a**	14.00 ± 1.04b	11.87 ± 2.04c
**Flavone**	Apigenin	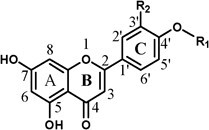		R1=H, R2=H	Api 7-*O*-Glc	433.11	1.74 ± 0.71c	1.66 ± 0.66c	**10.27 ± 1.40a**	3.08 ± 0.98b	
	Diosmetin			R1=CH3, R2=OH	Dio 7-*O*-Glc	463.12	ND	1.72 ± 0.12c	**6.63 ± 0.45a**	3.11 ± 0.86b	
	Acacetin			R1=CH3, R2=H	Aca 7-*O*-Glc	447.13	0.91 ± 0.06b	0.18 ± 0.02c	**1.88 ± 0.89a**	0.86 ± 0.11b	
**Flavonol**	Kaempferol	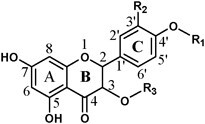		R1=H, R2=H, R3=H	Kae 7-*O*-Glc	449.11	1.67 ± 0.69b	0.82 ± 0.18b	ND	**9.26 ± 1.45a**	
	Quercetin			R1=H, R2=OH, R3=H	Que 7-*O*-Glc	465.10	ND	1.02 ± 0.15b	**38.81 ± 6.65a**	ND	

a The product content after 60 min of reaction with 20 μg of protein at a substrate concentration of 200 μM.

b Multiple comparisons of the flavonoid 7-*O*-glucoside product contents catalyzed by four 7GlcT proteins under the same substrate conditions. Bold font indicates the enzyme with the highest level of catalytic activity towards the substrate. All data are presented as mean ± SE from three independent replicate assays.

### Four functional Cg7GlcT proteins exhibit different substrate preference

The optimal pH and temperature for the *in vitro* catalytic reaction of four Cg7GlcTs were determined by using naringenin as a substrate (Fig. S7). The results showed that CgUGT89D30 exhibited the highest enzymatic activity at pH 7.0 (in 50 mM PBS buffer) and temperature of 30°C. Nevertheless, CgUGT90A31, CgUGT89AK1, and CgUGT73AC12 displayed equivalent highest enzymatic activities at pH of 7.0 and temperature of 35°C.

To investigate the preference of Cg7GlcT recombinants for flavonoid substrates, their kinetic properties were determined using the Michaelis-Menten equation under optimal reaction conditions (Table 2, Fig. S8). Among the different tested flavonoid substrates, CgUGT89AK1 exhibited the most notable catalytic efficiency towards quercetin with a *K*cat/*K*m value of 1352.49 M^-1^s^-1^, followed by diosmetin and eriodictyol, with *K*cat/*K*m valued of 284.12 M^-1^s^-1^ and 154.19 M^-1^s^-1^, respectively. Hesperetin had the lowest *K*cat/*K*m values (16.64 M^-1^s^-1^). For CgUGT89D30, it exhibited the greatest catalytic efficiency for the flavone compound of apigenin, with a *K*cat/*K*m value of 88.23 M^-1^s^-1^. Notably, CgUGT90A31 exhibited greater catalytic efficiency towards flavanones, with *K*cat/*K*m values of 52.02 M^-1^s^-1^ for naringenin and 98.31 M^-1^s^-1^ for isosakuranetin. CgUGT73AC12 had a comparatively higher catalytic efficiency against isosakuranetin, with a *K*cat/*K*m value of 37.57 M^-1^s^-1^. Overall, CgUGT89AK1 exhibited a superior catalytic efficiency relative to other three Cg7GlcTs.

### Transient overexpression of *CgUGT90A31* and *CgUGT89AK1* enhances FG accumulation in pummelo leaves

**Figure 4:**
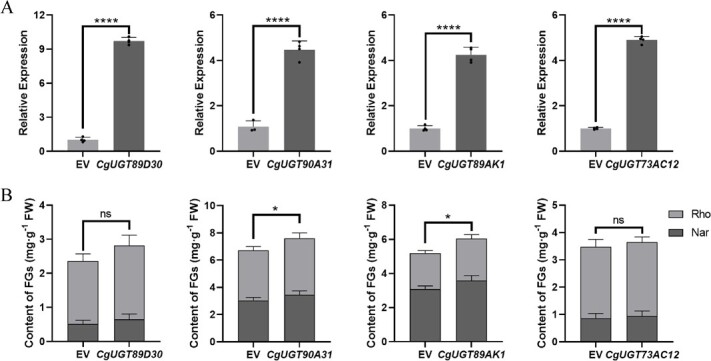
Effects of *Cg7GlcTs* transient overexpression on FG contents in ‘Huazhou’ pummelo leaves. (A) Expression pattern of *Cg7GlcTs* after *Cg7GlcTs*-pBI121 infiltration. (B) Content of FGs (mg g^−1^ FW, fresh weight) in the leaves of ‘Huazhou’ pummelo. Error bars represent SE from four biological replicates. Nar, naringin; Rho, rhoifolin. Student’s paired t-test. ns, not significant, ^*^P < 0.05, ^****^P < 0.0001.

‘Huazhou’ pummelo leaves are known to have abundant FGs^14^. Hence, transient overexpression was conducted in ‘Huazhou’ pummelo leaves to investigate the potential role of Cg7GlcTs in the glucosylation of flavonoids *in vivo*. The results demonstrated considerable upregulation of all four *Cg7GlcTs* in ‘Huazhou’ pummelo leaves (Fig. 4A). However, only those leaves treated with the bacterial solution containing *CgUGT90A31*-pBI121 and *CgUGT89AK1*-pBI121 exhibited significant increases of 1.13 and 1.16 folds in the overall content of FGs (naringin and rhoifolin), respectively (Fig. 4B).

**Table 2:** Kinetic parameters of four Cg7GlcTs

**Enzyme**	**Types**	**Substrate** ^**a**^	***K***_**m**_ **(μM)**	***V***_**max**_ **(μmol·S**^**-1**^**·mg**^**-1**^**)**	***K***_**cat**_ **(M**^***-1***^**)**	***K***_**cat**_ **/*K***_**m**_ **(S**^**-1**^**M**^**-1)**^
**CgUGT89D30**	Flavonone	Naringenin	79.57 ± 5.51	0.15 ± 0.00	0.09	18.11
		Hesperetin	207.41 ± 77.33	0.24 ± 0.05	0.14	11.00
		Isosakuranetin	261.00 ± 12.67	1.22 ± 0.03	0.69	43.73
		Eriodictyol	ND^b^	ND	ND	ND
	Flavone	Apigenin	17.34 ± 10.85	0.16 ± 0.02	0.09	**88.23** ^**c**^
		Diosmetin	ND	ND	ND	ND
	Flavonol	Kaempferol	35.76 ± 18.07	0.08 ± 0.01	0.05	21.92
		Quercetin	ND	ND	ND	ND
**CgUGT90A31**	Flavonone	Naringenin	139.04 ± 40.50	0.75 ± 0.10	0.43	52.02
		Hesperetin	179.10 ± 48.24	0.96 ± 0.12	0.55	51.38
		Isosakuranetin	111.49 ± 23.66	1.14 ± 0.10	0.66	**98.31**
		Eriodictyol	249.79 ± 15.61	1.30 ± 0.04	0.75	49.90
	Flavone	Apigenin	252.03 ±130.33	0.19 ± 0.05	0.11	7.14
		Diosmetin	34.93 ± 10.80	0.06 ± 0.00	0.03	15.76
	Flavonol	Kaempferol	56.20 ± 19.14	0.04 ± 0.00	0.02	6.48
		Quercetin	ND	ND	ND	ND
**CgUGT89AK1**	Flavonone	Naringenin	52.88 ± 17.30	0.27 ± 0.03	0.15	48.28
		Hesperetin	85.75 ± 14.53	0.15 ± 0.01	0.08	16.64
		Isosakuranetin	200.84 ± 17.92	0.95 ± 0.04	0.54	45.55
		Eriodictyol	26.25 ± 5.65	0.41 ± 0.02	0.24	154.19
	Flavone	Apigenin	24.37 ± 5.03	0.26 ± 0.01	0.15	107.06
		Diosmetin	6.16 ± 2.64	0.18 ± 0.01	0.11	284.12
	Flavonol	Kaempferol	ND	ND	ND	ND
		Quercetin	19.44 ± 11.00	2.77 ± 0.38	1.58	**1352.49**
**CgUGT73AC12**	Flavonone	Naringenin	105.34 ± 35.62	0.07 ± 0.01	0.04	6.36
		Hesperetin	169.68 ± 54.77	0.10 ± 0.01	0.06	5.62
		Isosakuranetin	363.99 ± 43.13	1.43 ± 0.10	0.82	**37.57**
		Eriodictyol	ND	ND	ND	ND
	Flavone	Apigenin	153.66 ± 62.17	0.10 ± 0.02	0.05	5.93
		Diosmetin	ND	ND	ND	ND
	Flavonol	Kaempferol	92.58 ± 55.02	0.11 ± 0.02	0.06	11.00
		Quercetin	ND	ND	ND	ND

a 1mM UDP-glucoside was used as sugar donor.

b ND, not determined due to the low activity.

c Bold font indicates the highest catalytic efficiency of enzymes towards flavonoid substrates.

All data are presented as mean ±SE from three independent replicate assays.

### Homologues of *CgUGT90A31* and *CgUGT89AK1* exhibit similar glucosylation function in other citrus species

**Figure 5:**
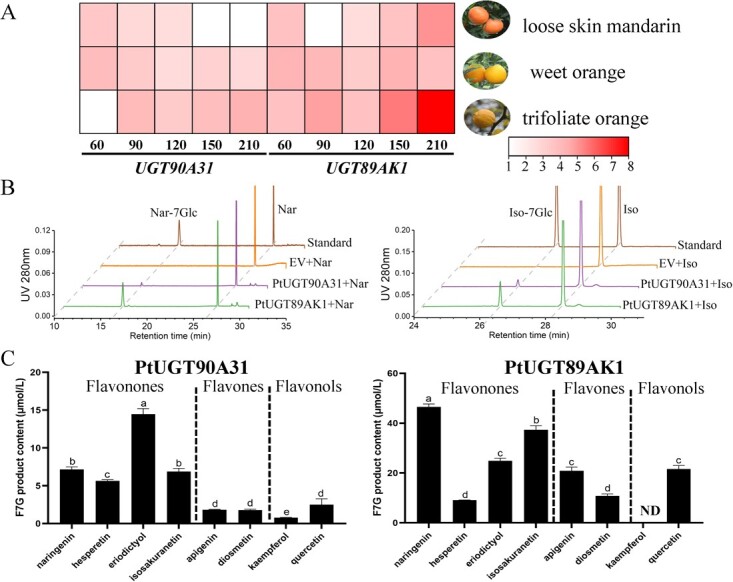
Expression patterns and functional activities of *UGT90A31* and *UGT89AK1* in citrus. (A) Heat map of the expression patterns of *UGT90A31* and *UGT89AK1* genes in the pulp of three citrus germplasms during development. The color scale on the right represents the FPKM values. The results are averages of FPKM of three biological replicates. (B) HPLC chromatograms of glucosylation of naringenin and isosakuranetin catalyzed by PtUGT90A31 and PtUGT89AK1 *in vitro*. Nar, naringenin; Nar-7Glc, naringenin 7-*O*-glucoside; Iso, isosakuranetin; Iso-7Glc, isosakuranetin 7-*O*-glucoside. (C) F7G product content under catalysis by PtUGT90A31 and PtUGT89AK1 on three groups of flavonoid substrates: flavonones, flavones, and flavonols. F7G, flavonoid 7-*O*-glucoside. Values are means ± SE (n = 3); ND, not detected.

We performed a genetic variability analysis of citrus *UGT90A31* and *UGT89AK1* across several germplasms from loose skin mandarins (*C. reticulata* ‘Chachiensis’), sweet orange (‘Fengjie 72-1’ Navel Orange), and trifoliata orange. The results demonstrated that both two genes exhibited high degrees of conservation in sequences (Fig. S9). Subsequently, we obtained the transcriptome data from fruit pulp of the three selected cultivars. The data showed that the expression level of *UGT90A31* was higher at the initial developmental stages of loose skin mandarins and sweet orange, whereas at the mature stage of trifoliata orange (Fig. 5A). On the contrary, the expression level of *UGT89AK1* significantly increased at the later developmental stage of loose skin mandarins and trifoliata orange pulp. However, in sweet orange pulp, the levels of *UGT89AK1* were comparatively stable throughout the development.

We further carried out sequence analysis among various species. The results revealed that PtUGT90A31 and PtUGT89AK1 exhibited the most significant sequence divergence from CgUGT90A31 and CgUGT89AK1 in trifoliata orange (Fig. S9). A total of eight flavonoids, including four flavanones, two flavones, and two flavonols, were selected as substrates to evaluate the activity of PtUGT90A31 and PtUGT89AK1. The HPLC results revealed that PtUGT90A31 and PtUGT89AK1 can catalyze naringenin and isosakuranetin, two main flavonoid aglycones, in the formation of corresponding 7-*O*-glucosides in *Poncirus trifoliata* (Fig. 5B). Among them, PtUGT90A31 exhibited the highest catalytic activity for isosakuranetin, while PtUGT89AK1 had the strongest catalytic activity for naringenin, followed by isosakuranetin (Fig. 5C).

### *CitUGT89AK1* may be involved in the stress response of citrus to HLB infection

**Figure 6:**
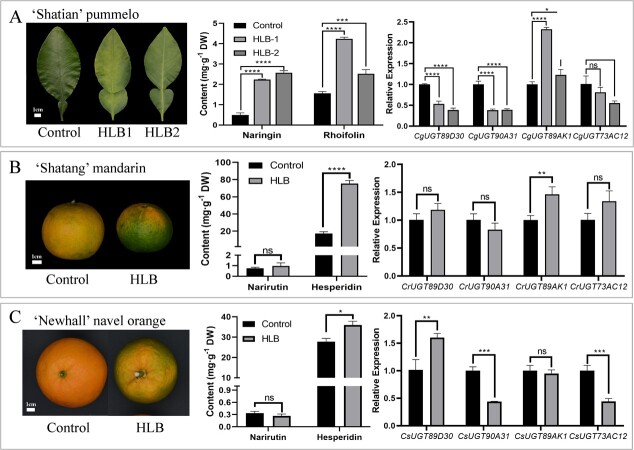
Changes in phenotype, content of main FGs, and expression levels of *Cit7GlcTs* in citrus under HLB infection. (A), (B), and (C) show the changes in phenotype, content of main FGs, and expression levels of *Cit7GlcTs* after HLB infection in ‘Shatian’ pummelo, ‘Shatang’ mandarin, and ‘Newhall’ navel orange, respectively. HLB, Huanglongbing. ns, not significant, ^*^P < 0.05, ^**^P < 0.01; ^***^P < 0.001; ^****^P < 0.0001.

Flavonoids have been documented to play an important role in modulating plant stress response [32]. Citrus HLB is the most devastating citrus disease worldwide. After infection with HLB, biological replicate 1 and biological replicate 2 of ‘Shatian’ pummelo showed leaf mottle, and all exhibited significant increases (averagely 2.82 folds) in the content of FGs (naringin and rhoifolin) in comparison with the control leaves, accompanied by substantial upregulation of *CgUGT89AK1* (averagely 1.74 folds) and downregulation of the other three *Cg7GlcTs* (Fig. 6A). For loose skin mandarins, HLB infection led to uneven fruit coloring, and significantly increased the content of the major FG hesperidin in ‘Shatang’ mandarin fruit peel (about 4.35 folds) along with significant upregulation of *CrUGT89AK1* (1.46 folds) compared with the control fruit (Fig. 6B). After being infected with HLB, ‘Newhall’ navel orange fruits also exhibited uneven coloring. The content of the main FG hesperidin was significantly increased, but the significantly upregulated gene was *CsUGT89D30* instead of *CsUGT89AK1* (Fig. 6C).

Furthermore, there was no significant change in the content of FGs in ‘Hybrid’ pummelo leaves after infection by citrus canker. However, a 1.5-fold increase in the transcript level of *CgUGT90A31* was still observed (Fig. S10 A). Moreover, in the leaves of ‘Newhall’ navel oranges infected with citrus canker, no significant changes in both FG contents and gene expression levels were observed (Fig. S10 B).

## Discussion

### Citrus flavonoid 7-*O*-glucosyltransferases are encoded by multiple *UGT* genes

As a superfamily of genes in plant flavonoid metabolism pathways, the *UGT* genes are often subjected to glycosylation modification [25, 33]. In *Lotus japonicus* seeds, it was observed that the production of flavonol glucosides is due to the collaborative effect of at least three genes in the UGT72 family [34], while in *Pueraria lobata*, three PlUGT recombinant enzymes could glucosylate the hydroxyl groups at the 7-position of genistein and daidzein isoflavones *in vitro*, with PlUGT15 demonstrating the highest catalytic efficiency [35]. In this study, four Cg7GlcTs exhibited catalytic activities towards the 7-hydroxy site of flavonoids *in vitro* (Fig. 3), as well as different affinities to nine flavonoid substrates (Table 2). Furthermore, the transcriptome analysis of two pummelo fruit demonstrated that *CgUGT89D30* and *CgUGT89AK1* have the most pronounced expression levels at the initial development stage of peel and pulp along with the highest FG content. However, the expression levels of *CgUGT90A31* and *CgUGT73AC12* were reached to peak during the initial development stage of ‘Huazhou’ pummelo peel and pulp, respectively (Fig. 2B). Interestingly, Shen et al. used mGWAS to analyze the content of melitidin, an acylated FG, in mature pummelo fruits and identified four *CgUGT* genes. Functional analysis showed that CgUGT1 and CgUGT2 have catalytic activities of 7-*O*-glucosylation only for flavones, while CgUGT3 and CgUGT4 have stronger 7-*O*-glucosylation activities for flavanones and flavones, the expression patterns of four *CgUGTs* varied across different citrus tissues [28]. Therefore, it can be speculated that several *7GlcTs* jointly regulate the biosynthesis of FGs in citrus, which can ensure the production of flavonoid 7-*O*-glucoside derivatives across different citrus tissues and developmental stages.

### *CitUGT89AK1* may be a key gene for boosted FG biosynthesis in citrus fruits

Although Cit7GlcTs have been isolated in the 1990s from grapefruit (*C. paradisi*) [36] and lemon (*C. lemon*) [37] cell-free extracts, the genes encoding flavonoid *7GlcTs* were rarely reported in the last two decades. In the present study, we successfully discovered four Cg7GlcTs from pummelo. Unlike CsUGT76F1, which has been previously reported to favor catalyzing flavonol substrates, the four Cg7GlcTs exhibit higher catalytic activity in glucosylating the 7-hydroxy position of flavanone and flavone substrates. However, CgUGT89AK1 showed enhanced catalytic efficiency specifically to quercetin. Among the four Cg7GlcTs, the recombinant enzyme CgUGT89AK1 exhibited notable affinity and catalytic efficiency towards flavonoid substrates *in vitro* (Table 1). Transient overexpressed four *Cg7GlcT* genes in ‘Huazhou’ pummelo leaves, in which the overexpression of *CgUGT90A31* and *CgUGT89AK1* promoted the FG contents. While *CgUGT89D30* and *CgUGT73AC12* have not significantly affected the FG contents in pummelo leaves due to their weak catalytic activity towards the flavonoid 7-hydroxy position (Fig. 4). Furthermore, we selected the homologous sequence PtUGT89AK1, a candidate with the highest sequence divergence from CgUGT89AK1, for functional validation in other citrus species. The results showed that PtUGT89AK1 could proficiently catalyze a diverse range of flavonoid substrates (Fig. 5), implying the consistent functions of CitUGT89AK1 in various citrus species.

In addition, ‘Huazhou’ pummelo is a source material for the precious traditional Chinese medicine ‘Huajuhong’, which is characterized by particularly high accumulation of naringin that contributes to its medical value [38]. Compared with the commonly cultivated ‘Fenghuang’ pummelo, ‘Huazhou’ pummelo fruits showed higher FG contents throughout the entire development period, which was even 4-fold that of ‘Fenghuang’ pummelo in the pulp at 60 DAF (Fig 1C). Accordingly, the expression level of *CgUGT89AK1* in the 60 DAF pulp of the ‘Huazhou’ pummelo was 6-fold higher than that in ‘Fenghuang’ pummelo. However, no significant difference was observed in the peel, which is consistent with the early FG metabolism in the ‘Huazhou’ pummelo. Therefore, we speculate that the expression level of UGT89AK1 may be responsible for the difference in FG content among different citrus cultivars. Collectively, it can be speculated that *CitUGT89AK1* may be a promising gene in citrus fruits that can be utilized to improve FG production.

### *CitUGT89AK1* may participate in the stress response of citrus to HLB infection by promoting flavonoid synthesis

Citrus HLB caused by the infection of ‘*Candidatus* Liberibacter spp.’ is the most devastating citrus disease worldwide [39], which can seriously affect the yield, fruit quality, and life span of citrus. There have been no effective treatments to date, but the disease can be efficiently alleviated by some special measures. It has been demonstrated that enhancing the synthesis ability of antioxidants such as flavonoids and inhibiting the generation of reactive oxygen species is an effective method for controlling HLB [17]. In a previous study, the leaves of Hamlin sweet orange and Valencia orange infected with HLB showed more than ten folds of increases in flavonoid content [40]. It was found that nine flavonoids, including vitexin, cosmetin (apigenin 7-*O*-glucoside), and rhoifolin, exhibited considerable increases at the early stage of citrus leaves infected with HLB [18]. A recent study has indicated that rutin has the potential to mitigate the detrimental effect associated with HLB [41].

In this study, we observed a considerable rise in the FG content in two tissues infected with HLB in ‘Shatian’ pummelo and ‘Shatang’ mandarin. Simultaneously, there was observable upregulation of *CitUGT89AK1*, suggesting that it is likely involved in the response of citrus to HLB by facilitating the synthesis of downstream FGs. In contrast, in ‘Newhall’ navel orange fruits infected with HLB, the expression level of *CsUGT89AK1* remained unchanged, while *CsUGT89D30* was significantly upregulated by 1.5 folds. Compared with other citrus germplasm infected with HLB, ‘Newhall’ navel orange only showed slight increases in FG content, which may be due to the unchanged expression of *CsUGT89AK1*. Therefore, subsequent research can focus on the binding elements related to stress response in the promoter region of *CitUGT89AK1*. It is worth noting that HLB invasion is rarely found in the ‘Huazhou’ pummelo production area, indicating the presence of a tolerant phenotype associated with the accumulation of FGs. Furthermore, after citrus canker infection of the tissues of ‘Hybrid’ pummelo and ‘Newhall’ navel orange, no significant change was observed in the FG contents, despite the upregulation of *CgUGT90A31* in ‘Hybrid’ pummelo. This result indicate that CgUGT90A31 plays a weaker role in 7-hydroxy position glucosylation than CgUGT89AK1, though it might be involved in the response of citrus to canker infection.

## Materials and methods

### Plant materials

The information on the 10 citrus accessions used in this study is listed in Table S2. For fruits at different development stages, uniform ‘Huazhou’ pummelo (*C. grandis*) and ‘Fenghuang’ pummelo (*C. grandis*) fruits without mechanical damage were harvested at 30, 60, 90, 120, 150 and 210 DAF. ‘Chachiensis’ (*C. reticulata*) and trifoliata orange (*P. trifoliata*) fruits without mechanical damage were harvested at 60, 90, 120, 150 and 210 DAF. The fruits of other citrus germplasms were collected at maturity. Except for 30 DAF (no pulp tissue was developed at the stage, and thus the whole fruit was investigated as the peel), the fruits of the above plant materials were divided into two parts, peel (including flavedo and albedo) and pulp (including segment membrane and juice sacs) tissues. At each stage, 15–25 fruits were harvested. In addition, for citrus leaves, thirty healthy and fully expanded adult leaves were collected. All samples were randomly divided into three biological replicates and washed with water, then promptly immersed in liquid nitrogen, and stored at −80°C until further analysis.

### Chemical standards and reagents

Twenty-seven flavonoid standards, including sixteen flavonones (naringenin, hesperetin, isosakuranetin, eriodictyol, prunin, hesperetin 7-*O*-glucoside, isosakuranetin 7-*O*-glucoside, eriodictyol 7-*O*-glucoside, eriocitrin, narirutin, hesperidin, didymin, neoeriocitrin, naringin, neohesperidin, and poncirin), seven flavones (apigenin, diosmetin, acacetin, apigenin 7-*O*-glucoside, diosmetin 7-*O*-glucoside, acacetin 7-*O*-glucoside, rhoifolin), and four flavonols (quercetin, kaempferol, quercetin 7-*O*-glucoside, kaempferol 7-*O*-glucoside) were provided by YuanYe Biotechnology Ltd (Shanghai, China) and dissolved in dimethyl sulfoxide at a concentration of 10 mg mL^-1^ or 20 mmol L^-1^ as stock solutions. UDP-glucose standards were purchased from Sigma-Aldrich, while acetonitrile and methanol were purchased from Thermo Fisher Scientific (Waltham, MA, USA), and formic acid was purchased from Aladdin (Shanghai, China). Deionized water was prepared from a Milli-Q ULTRA purification system (Millipore, Billerica, MA, USA).

### Extraction, identification, and quantification of FGs

Extraction of FGs were performed as described in our previous study [22]. Citrus fruit and leaf samples were ground into powder under liquid nitrogen and then lyophilized using Heto Lylab 3000 (Heto-Holten A/S, Allerød, Denmark). Around 0.1 g of powdered was sonicated in 5 mL of 70% aqueous methanol for 1 hour in an DTC-15J ultrasonic cleaning machine (Toptll Technology, Hubei, China). After centrifugation at 8000 rpm for 10 min, supernatant was extracted and filtered through a 0.22 μm micropore membrane filter for LC-MS or HPLC analysis.

FGs were determined according to Wang et al [42]. Mass spectrometry data were acquired using a 1200 Series Rapid Resolution UPLC system coupled with a 1260 infinity array detector and a 6520 accurate-Mass Quadrupole Time-of-Flight (Q-TOF) MS system (Agilent Technologies, CA, USA). Separation of flavonoids was conducted using a Shim-pack VP-ODS C18 (Shimadzu, Kyoto, Japan). Ultrapure water and acetonitrile containing 0.04% (v/v) formic acid were utilized as mobile phases A and B, respectively. Raw data were underwent processing using Agilent MassHunter Qualitative Analysis B.04.00 software. FGs were identified by comparing retention times, UV spectra, mass spectra, and ion fragments between the samples and flavonoid standards. The identities of compounds without standards can be speculated based on their molecular weight and ion fragments.

FGs were quantified utilizing an HPLC system consisting of a 1525 Binary HPLC pump, a 2717 Autosampler and a 2998 Photodiode Array Detector (Waters, Milford, MA). Flavonoids were separated via a C18 Hypersil GOLD column (Thermo Fisher Scientific, Waltham, MA). Mobile phases A and B comprised ultrapure water and acetonitrile containing 0.15% (v/v) formic acid, respectively. The gradient elution system and instrument conditions were consistent with those described by Peng et al [43]. The ultraviolet-visible spectra were recorded within the range of 210 to 400 nm. Quantification of the flavonoid 7-*O*-glucosides were performed using the calibration curves made with the corresponding commercial standards (Table S3). The flavonoid disaccharide calibration curves refer to Chen et al [22].

### Phylogenetic analyses and sequence alignment

The PSPG box was taken as a query according to Wu et al [44]. A BLASTP search of the pummelo genome (http://citrus.hzau.edu.cn/index.php) was performed using the UGT conserved sequence. HMM file (PF00201, UGT. HMM) for the conserved domain hidden Markov model of the *UGT* gene family was downloaded from Pfam (http://pfam.xfam.org/). The amino acid sequences of predicted UGTs were used for phylogenetic analysis. Different protein sequences were aligned using the ClustalW program, and the phylogenetic tree was constructed using MEGA 7 software (Mega Software, State College, PA, USA). The construction of a phylogenetic tree was based on the Neighbor-Joining statistical method and was tested with 1000 bootstrap replicates. Genbank accession numbers of sequences employed in the study can be found in supplemental Table S4.

### RNA extraction, RNA-seq, and qRT-PCR analysis

The total RNA was isolated using an EASYspin Plus Plant RNA Kit (Aidlab, Beijing, China). RNA was reverse transcribed into cDNA with HiScript II 1st Strand cDNA Synthesis Kit (+gDNA wiper) (Vazyme, Nanjing, China). A total of 66 RNA-seq libraries from the peel and pulp of ‘Huazhou’ pummelo and ‘Fenghuang’ pummelo at six developmental stages (30, 60, 90, 120, 150, and 210 DAF) were generated, and correlation between FG contents and FPKM values of *7GlcTs* were performed using Pearson correlation coefficient. In addition, transcriptome data from five developmental stages (60, 90, 120, 150, and 210 DAF) of ‘Chachiensis’ and trifoliata orange pulp tissue were obtained for gene expression level analysis. ‘Fengji 72-1’ navel orange pulp transcriptome data were obtained from Feng et al [45].

qRT-PCR was performed using a LightCycler 480 (Roche, Switzerland) with the SYBR Green system (Yeasen, Shanghai, China), as described by Lu et al [46]. To verify the expression levels of *7GlcTs*, each qPCR was performed with three independent biological replicates. The primers are listed in Supplemental Table S5 and are based on Citrus Pan-genome (http://citrus.hzau.edu.cn/index.php) database gene sequences [47]. The *actin* gene was used as the reference control [48]. Expression of genes was calculated by the 2^-ΔΔCt^ method.

### Isolation, cloning, protein expression and purification of *7GlcTs*

The *7GlcT* genes were isolated based on the Citrus Pan-genome database. The full-length coding sequence (CDS) of *7GlcT* genes was amplified using the primers described in Table S5, and then inserted into the pMal-c2x vector, followed by transfer to *Escherichia coli* BL21 (DE3) for expression. Transformants carrying the expression plasmid *7GlcTs* were incubated to OD_600_=0.6 in Luria-Bertani (LB) medium containing 0.1 g L^-1^ ampicillin at 37°C, followed by the addition of isopropyl β-D-thiogalactopyranoside to a final concentration of 0.3 mM and then cultured at 16 °C after shaking at 130 rpm for 18 h. Protein purification was performed according to Li et al [49]. Subsequently, the protein was concentrated by ultrafiltration using 30 mL (50 kDa MWCO) Ultrafiltration Spin Columns (Millipore, Billerica, MA, USA). Purified proteins were evaluated by sodium dodecyl sulphate-polyacrylamide gel electrophoresis (SDS-PAGE).

### Assay of enzyme activity and kinetics

The enzyme activity assay was determined with reference to Peng et al [12]. The reaction total volume was 100 μL, containing phosphate buffered saline (50 mM, pH 7.3), 1 mM UDP-glucose, 5 mM MgCl_2_, 0.2 mM flavonoid substrates and 20 μg recombinant proteins. The reaction was incubated at 35 °C for 60 min and terminated by adding 200 μL methanol. Then, the resulting products were filtered through a 0.22 μm filter and analyzed by HPLC or LC-MS.

The optimal reaction conditions were determined by varying pH and temperature. Following the method described above, three distinct buffer solutions are employed to regulate pH levels for enzyme determination: acetic acid and sodium acetate buffer (pH 3.6–5.8), phosphate buffered saline (pH 6.0–8.0), and glycine and sodium hydroxide buffer (pH 8.6–10.6). The optimum reaction temperature was determined to be in the range of 25 °C to 60 °C in phosphate buffered saline (pH 7.3).

To determine the enzymatic kinetics for different candidate UGTs, recombinant proteins (10 μg) were added to reaction mixtures containing 50 mM phosphate buffered saline and 1 mM UDP-glucose in a final volume of 100 μL. Reactions were incubated at optimum reaction pH and temperature for 30 min, and the concentrations of tested flavonoid substrates ranged from 0 to 400 μM. All *in vitro* enzyme assays were repeated at least three times. Kinetic parameters were calculated using the Michaelis-Menten nonlinear curve fitting.

### Transient overexpression of *Cg7GlcTs* in citrus leaves

The transient overexpression assay was performed in citrus leaves as described by Hussain et al [50] with some modifications. Specifically, the CDS of *Cg7GlcTs* was inserted into the pBI121 vector. The *Cg7GlcTs*-pBI121 recombinants and the empty vector (as control) were transferred into *Agrobacterium tumefaciens* strain EHA105, respectively. The transformants harboring the recombinant vectors and the empty vector were initially cultured in LB Broth at 28°C. Then, the bacterial cells were separated by centrifugation and re-suspended to an OD_600_ of 0.7-0.8 in infiltration buffer (10 mmol L^-1^ MES, 10 mmol L^-1^ MgCl_2_, 200 μmol L^-1^ acetosyringone, pH 5.6 in equal volume). The suspensions containing the target genes and the control were injected separately into the lower epidermis of ‘Huazhou’ pummelo leaves on opposite sides. After five days, the treated leaves’ infiltrated regions were collected for subsequent analyses. FG contents and transcription levels of *Cg7GlcTs* were measured as described above. The primers utilized in constructing *Cg7GlcTs*-pBI121 and for the qRT-PCR analysis are provided in Table S5.

### Statistical analysis

Statistical analysis was performed using Microsoft Excel 2019. All data were obtained with at least three biological replicates, and error bars indicate standard errors (SE). Significant differences between the samples were analyzed with a two-tailed Student's t-test. Figures were produced with GraphPad Prism 8.0 (GraphPad SoftwareInc., San Diego, CA, USA) and OriginPro 2021 (Origin Lab Corporation, Northampton, USA). Structural formulae were drawn using ChemDraw ultra (PerkinElmer Informatics, Waltham, USA).

## Supplementary Material

Web_Material_uhae098

## Data Availability

The data used to support this study’s findings are included within the article. The RNA-seq data have been deposited in the NCBI Sequence Read Archive, accession number: PRJNA1072851 (http://www.ncbi.nlm.nih.gov/bioproject/1072851).
