# Supplementary material for: Four novel Cit7GlcTs functional in flavonoid 7-O-glucoside biosynthesis are vital to flavonoid biosynthesis shunting in citrus
Source: Hortic Res. 2024 Apr 25;11(6):uhae098. doi: 10.1093/hr/uhae098 (PMC11165160; doi:10.1093/hr/uhae098)
Supplement: Web_Material_uhae098 [file web_material_uhae098.zip › Supplementary Figures.pdf]

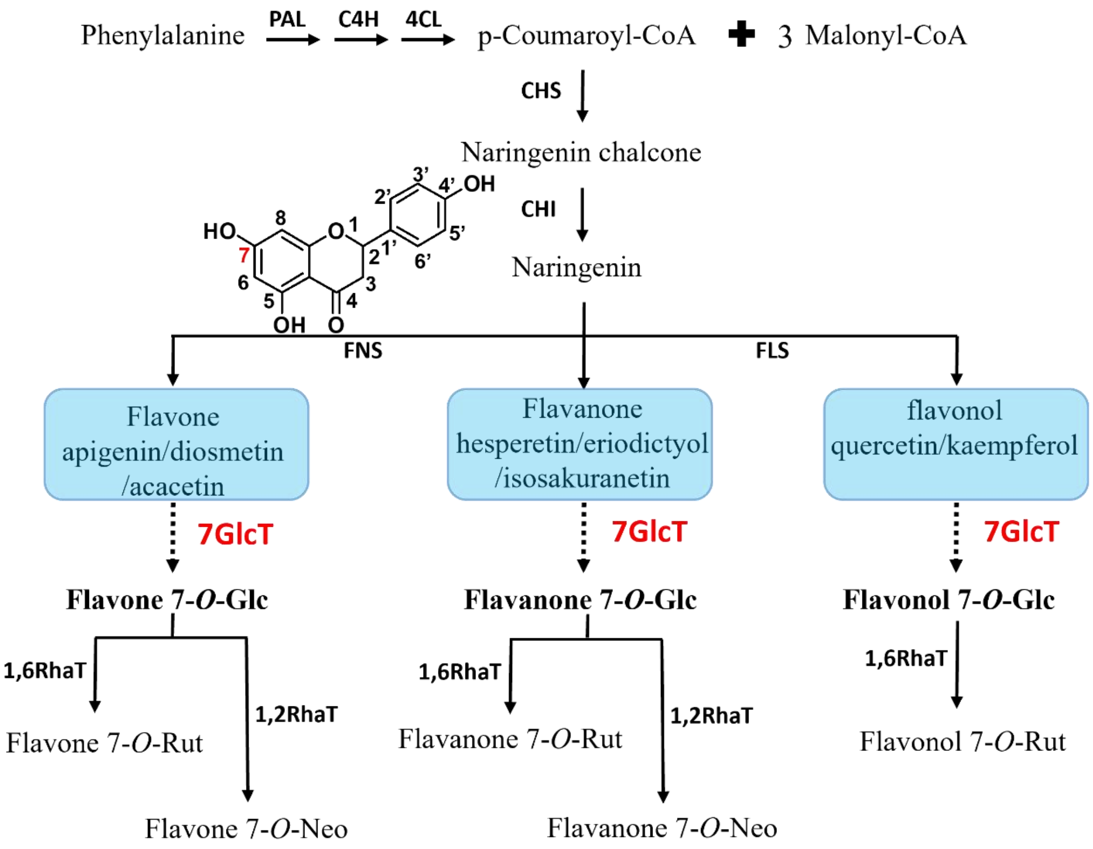

Fig. S1. FG biosynthetic pathway in citrus plants. PAL, phenylalanine ammonia-lyase; C4H, cinnamate-4-hydroxylase; 4CL, 4-coumarate: CoA ligase; CHS, chalcone synthase; CHI, chalcone isomerase; FNS, flavone synthase; FLS, flavonol synthase; 7GlcT, 7-*O*-glucosyltransferase; 1,6RhaT, 1,6-rhamnosyltransferase; 1,2RhaT, 1,2-rhamnosyltransferase; Glc, glucoside; Rut, rutinoside; Neo, neohesperidoside.

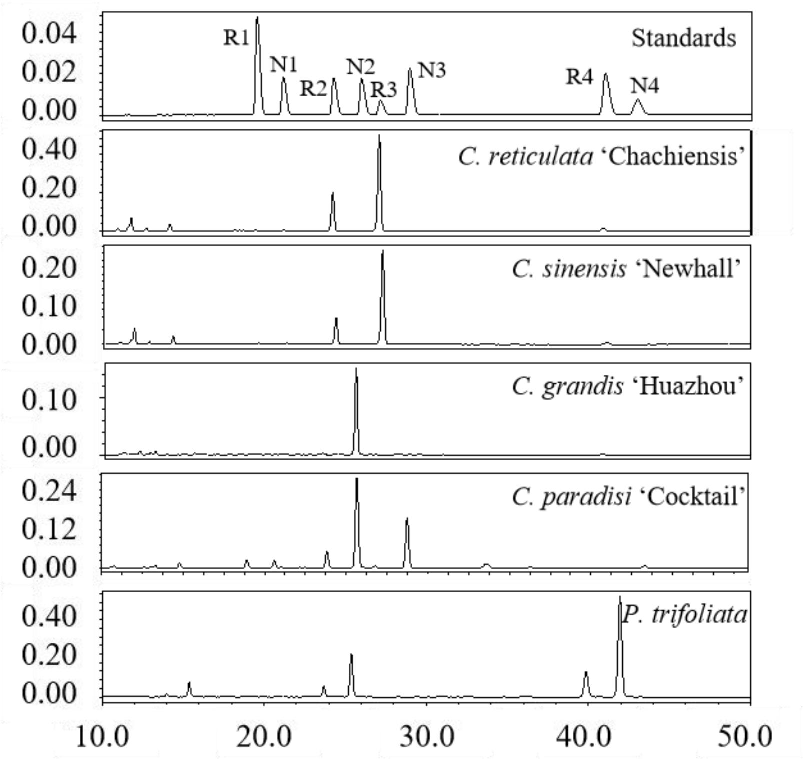

Fig. S2. HPLC analysis of FGs in mature fruit pulp of five representative citrus species based on chemical standards. R1−R4 stand for four rutinosides (eriocitrin, narirutin, hesperidin, and didymin, respectively); N1−N4 stand for four neohesperidosides (neoeriocitrin, naringin, neohesperidin, and poncirin, respectively).

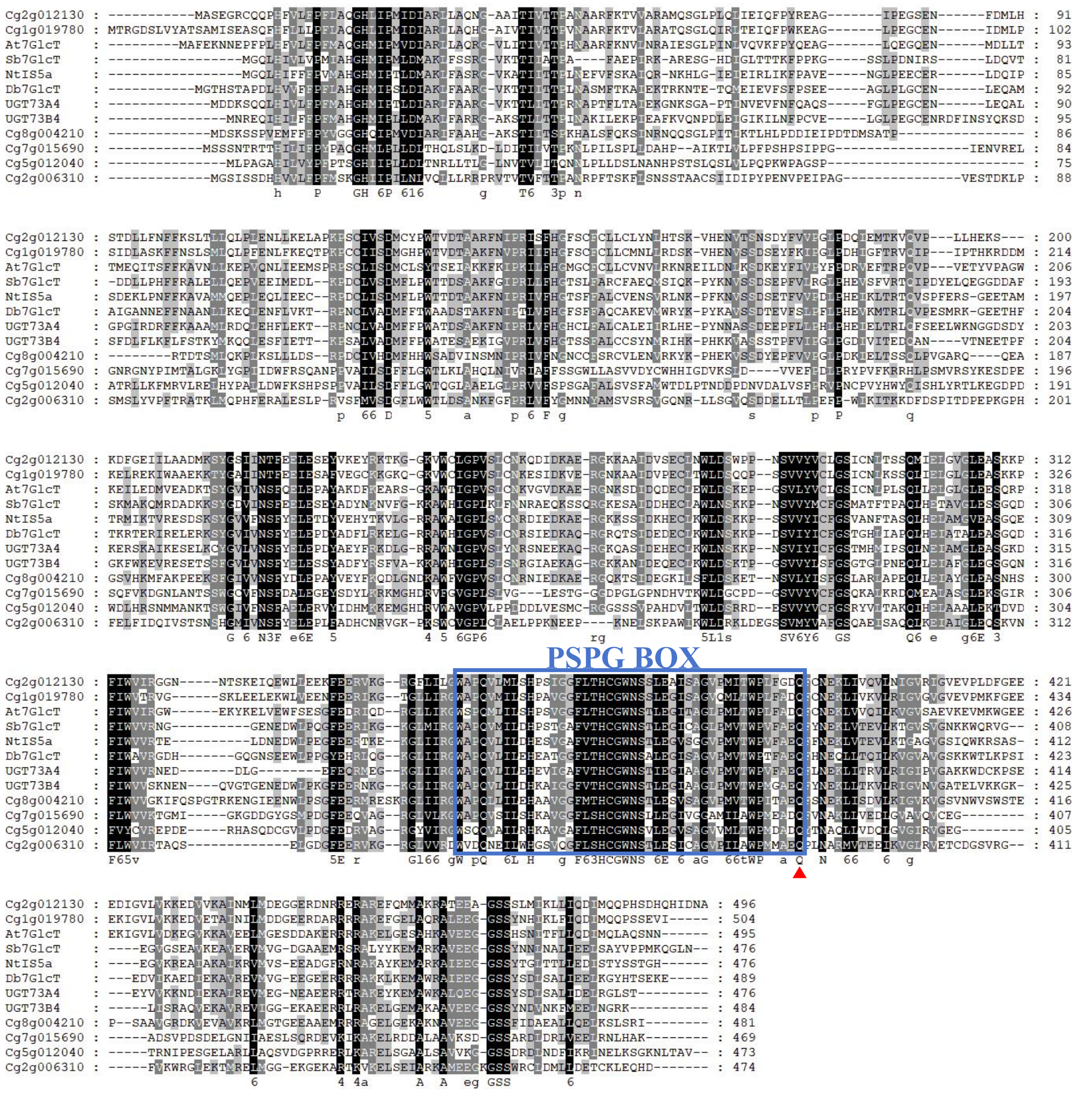

Fig. S3. Multiple sequence alignment of Cg7GlcTs with other known UGTs. The blue box is the PSPG conservative domain, red triangle marks the last amino acid Q in the PSPG box.

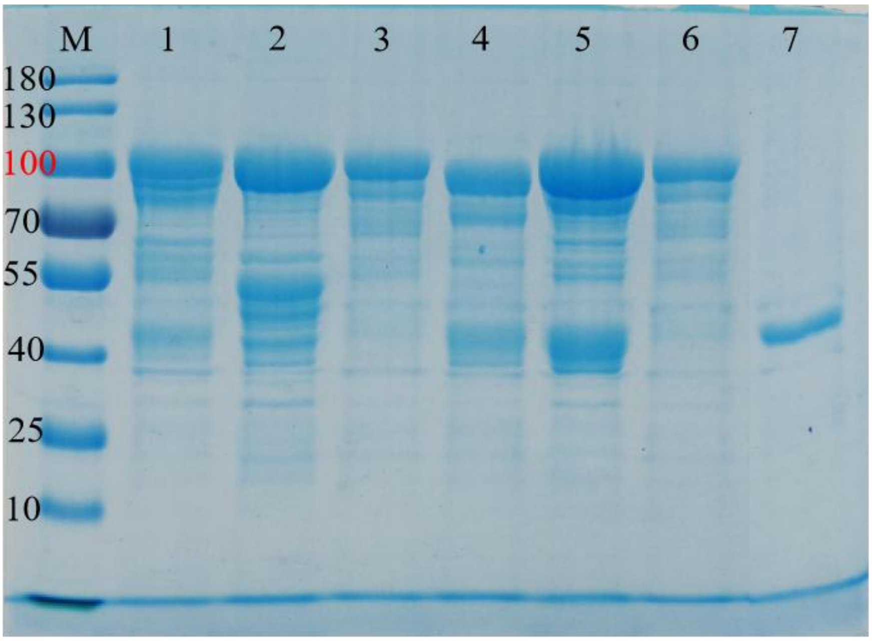

Fig. S4. SDS-PAGE analysis of the MBP-tagged Cg7GlcT proteins. M, protein molecular weight marker; Lane 1-6, Purified MBP-tagged CgUGT73CG30, CgUGT89D30, CgUGT90A31, CgUGT73CG31, CgUGT89AK1, and CgUGT73AC12 protein approximately 100 kDa, each consists of a 42.5 kDa maltose-binding protein and a Cg7GlcT protein of about 55 kDa; Lane 7, Purified MBP tagged (42.5 kDa maltose-binding protein).

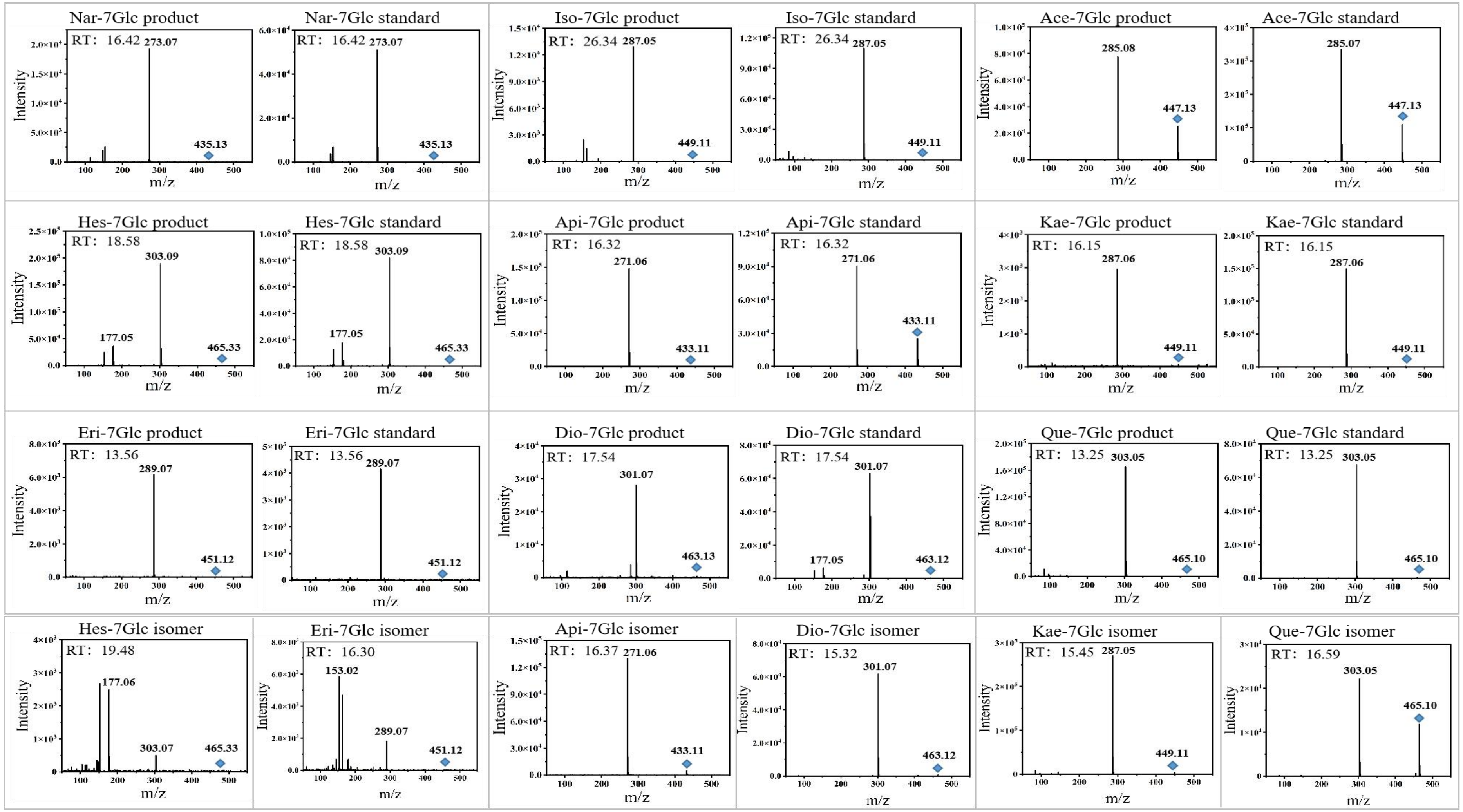

Fig. S5. MS/MS spectrometry of glucosylation products generated by Cg7GlcTs *in vitro* and corresponding authentic standards.

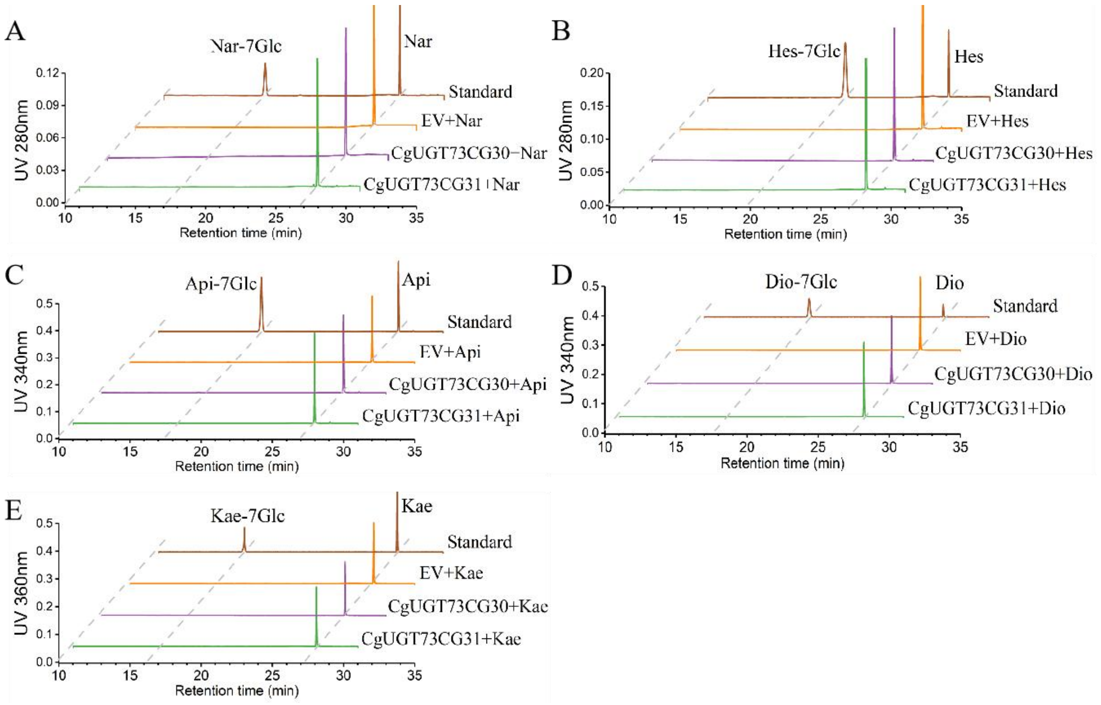

Fig. S6. HPLC chromatograms of glucosylation of various flavonoid substrates under the catalysis of CgUGT73CG30 and CgUGT73CG31 *in vitro*. (A) Nar, naringenin; Nar-7Glc, naringenin 7-*O*-glucoside; (B) Hes, hesperetin; Hes-7Glc, hesperetin 7-*O*-glucoside; (C) Api, apigenin; Api-7Glc, apigenin 7-*O*-glucoside; (D) Dio, diosmetin; Dio-7Glc, diosmetin 7-*O*-glucoside; (E) Kae, kaempferol; Kae-7Glc, kaempferol 7-*O*-glucoside. EV, empty vector.

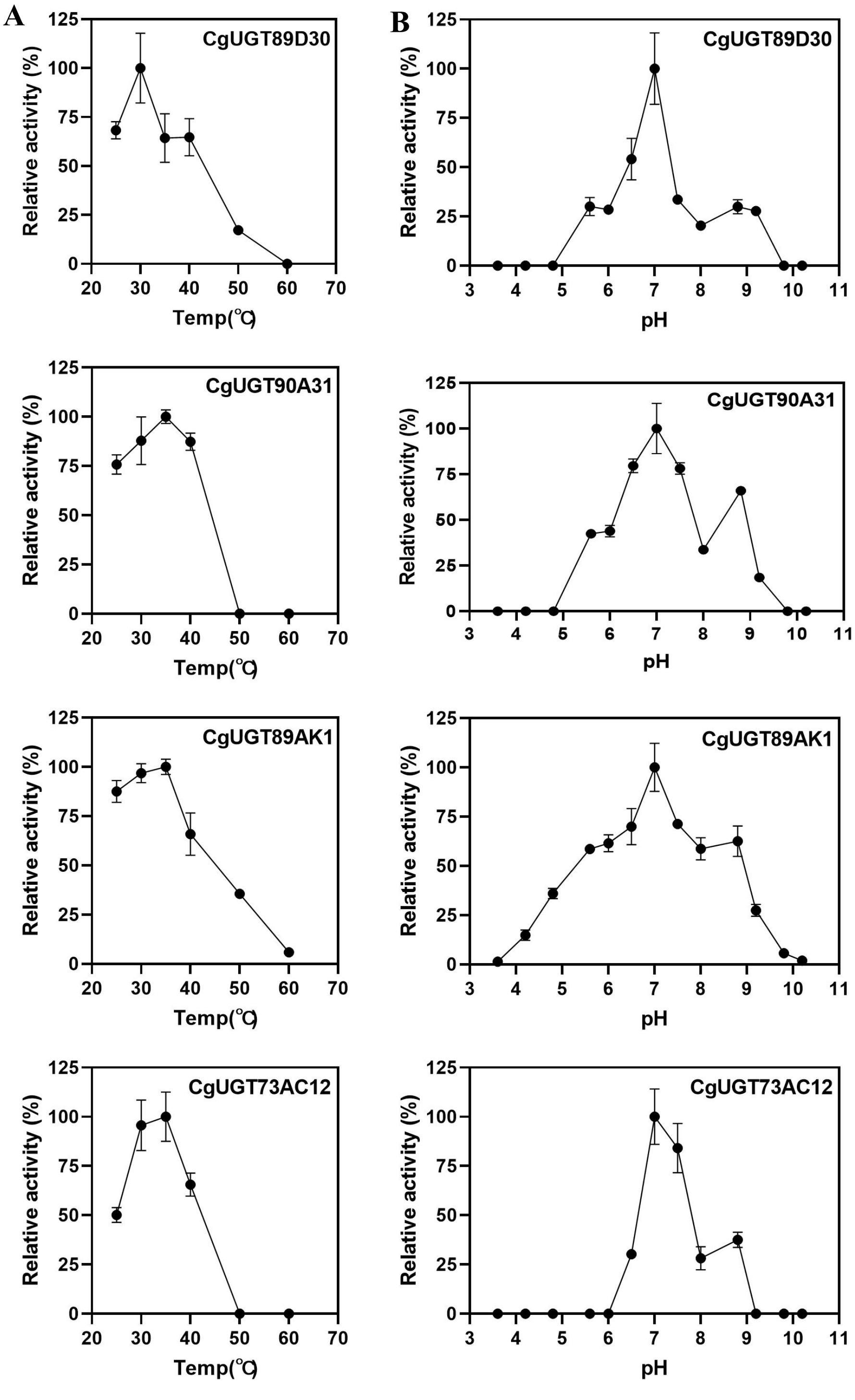

Fig. S7. Optimization of the reaction conditions of Cg7GlcTs. (A) Effect of reaction temperature on Cg7GlcTs activities, with temperature increments for 30 min at pH 7.3. (B) Effect of reaction pH on Cg7GlcTs activities, with pH increments for 30 min at 35°C. Assays were performed with naringenin as a substrate, and the peak area of its 7-*O*-glucosylation product prunin was used to determine the relative activity of the enzyme. Values are means ±SE (n=3).

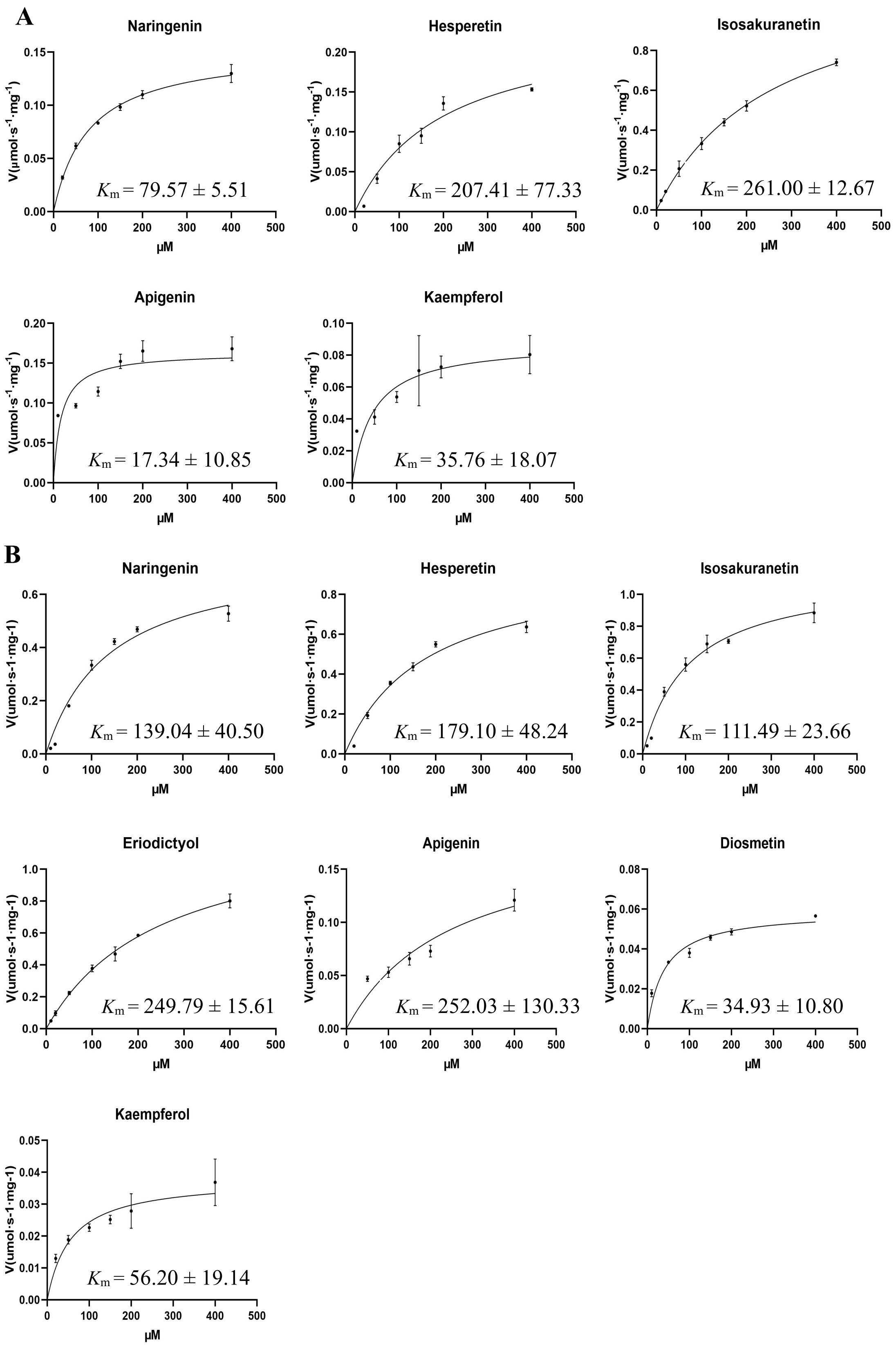
A
Naringenin
Km = 79.57 ± 5.51
Hesperetin
Km = 207.41 ± 77.33
Isosakuranetin
Km = 261.00 ± 12.67
Apigenin
Km = 17.34 ± 10.85
Kaempferol
Km = 35.76 ± 18.07
V(μmol·s-1·mg-1)
V(umol·s-1·mg-1)
μM
B
Naringenin
Km = 139.04 ± 40.50
Hesperetin
Km = 179.10 ± 48.24
Isosakuranetin
Km = 111.49 ± 23.66
Eriodictyol
Km = 249.79 ± 15.61
Apigenin
Km = 252.03 ± 130.33
Diosmetin
Km = 34.93 ± 10.80
Kaempferol
Km = 56.20 ± 19.14
V(umol·s-1·mg-1)
μM

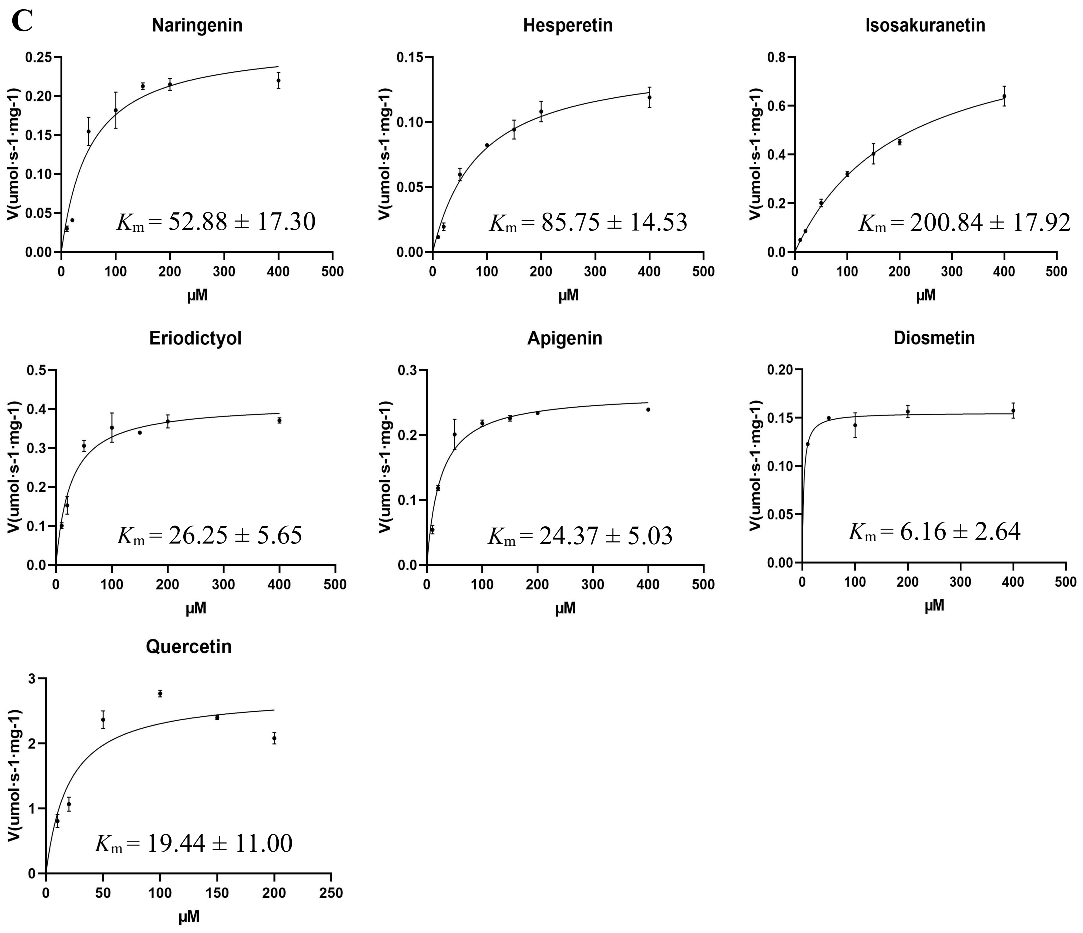

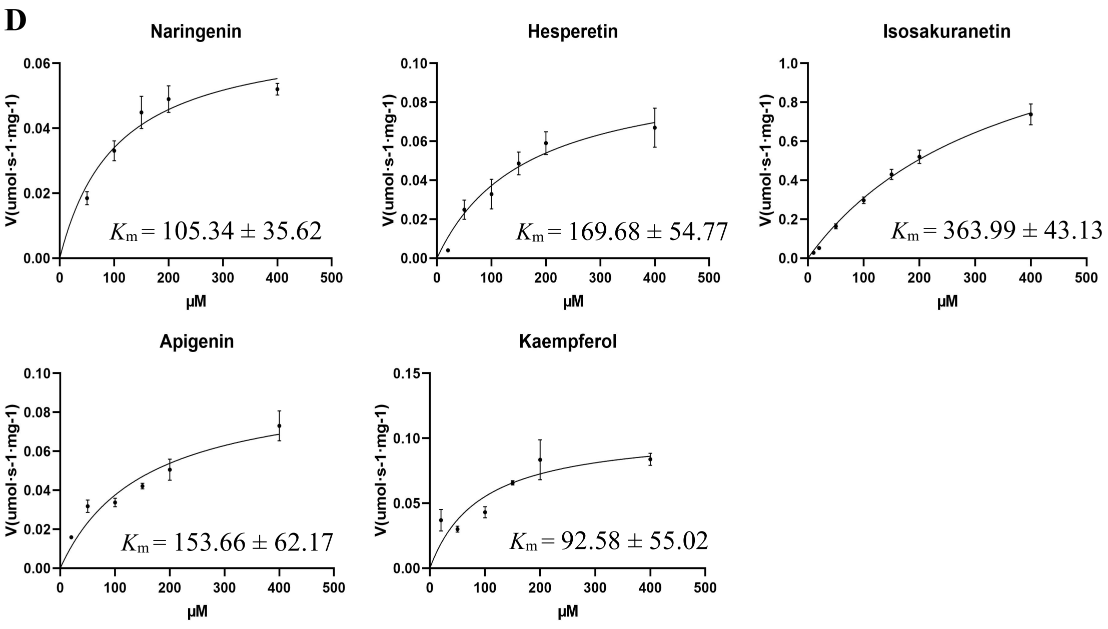

Fig. S8. Kinetic properties of Cg7GlcTs with different flavonoid substrates. Kinetic parameters were estimated by non-linear curve fitting using Michaelis–Menten. A, B, C, and D represent CgUGT89D30, CgUGT90A31, CgUGT89AK1, and CgUGT73AC12 recombinases, respectively.

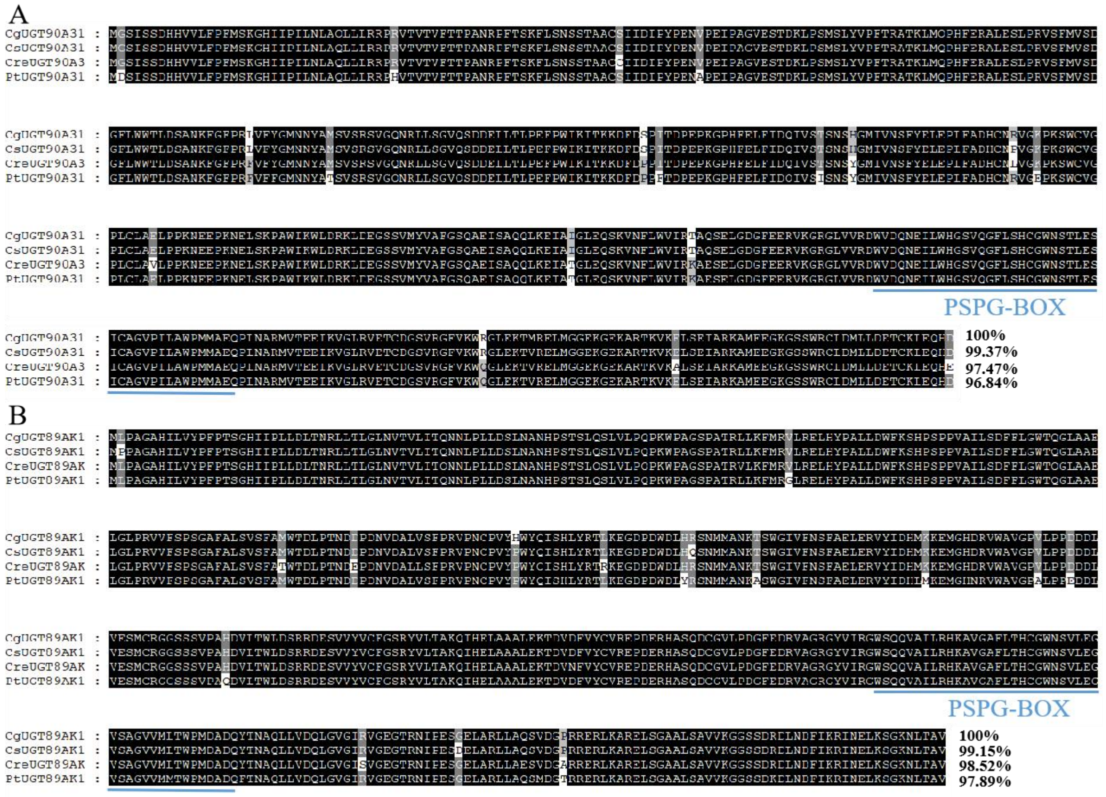

Fig. S9. Amino acid sequence alignment of UGT90A31 and UGT89AK1 in four citrus germplasms. (A) and (B) showed the sequence alignment results of UGT90A31 and UGT89AK1, respectively.

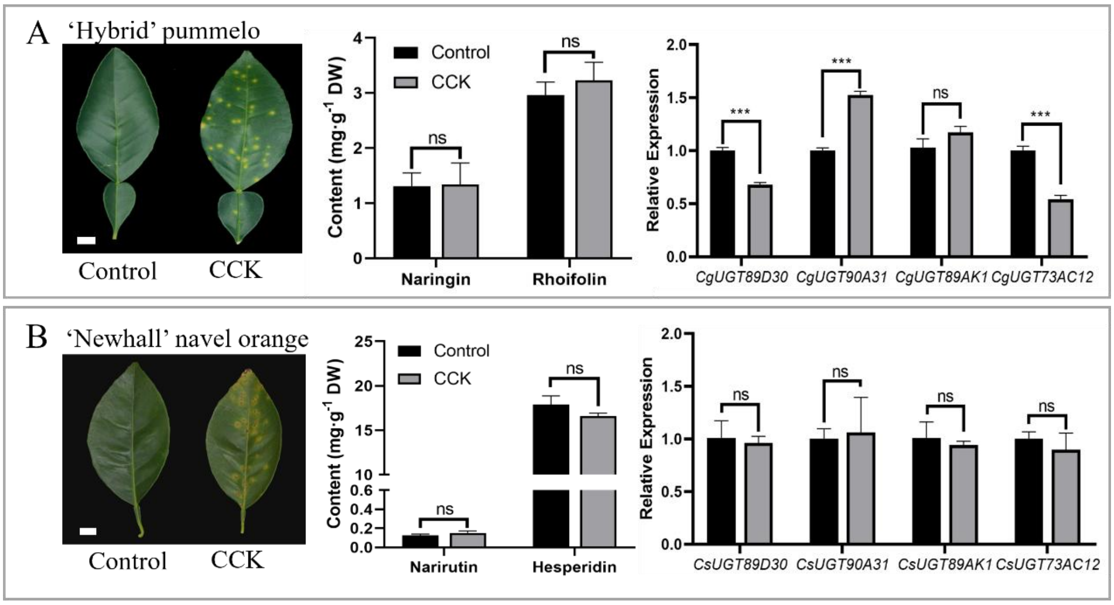

Fig. S10. Changes in phenotype, content of main FGs, and expression levels of *Cit7GlcTs* in citrus infected with citrus canker. (A) and (B) show the changes in phenotype, the content of main FGs, and the expression levels of *Cit7GlcTs* after CCK infection in 'Hybrid' pummelo and 'Newhall' navel orange, respectively. CCK, citrus canker. ns, not significant, ***$P < 0.001$.
